# Efficacy of Hank's balanced salt solution compared to other solutions in the preservation of the periodontal ligament. A systematic review and meta-analysis

**DOI:** 10.1371/journal.pone.0200467

**Published:** 2018-07-13

**Authors:** Nathalia Carolina Fernandes Fagundes, Leonardo Oliveira Bittencourt, Marcela Baraúna Magno, Márcia Martins Marques, Lucianne Cople Maia, Rafael Rodrigues Lima

**Affiliations:** 1 Laboratory of Functional and Structural Biology, Institute of Biological Sciences, Universidade Federal do Pará, Belém-Pará, Brazil; 2 School of Dentistry, Faculty of Medicine and Dentistry, University of Alberta, Edmonton, Canada; 3 Department of Pediatric Dentistry and Orthodontics, School of Dentistry, Universidade Federal do Rio de Janeiro, Rio de Janeiro, Brazil; 4 Department of Restorative Dentistry, School of Dentistry, Universidade de São Paulo, São Paulo, Brazil; Istanbul Universitesi Dis Hekimligi Fakultesi, TURKEY

## Abstract

This systematic review and meta-analysis (MA) aimed to verify the capacity of different storage media to preserve viability of periodontal ligament cells in comparison to Hank’s Balanced Salt Solution. The searches, selection process, data extraction and Risk of Bias control were conducted according to Preferred Reporting Items for Systematic Review and Meta-Analysis guidelines. Five MA were conducted to compare the cell viability between milk versus Hank's balanced salt solution (HBSS) in a dichotomous (1) or continuous (2) data model; tap water versus HBSS (3); medicinal herbals versus HBSS (4); and saline solution versus HBSS (5). 693 potentially studies were identified, with 18 studies included in the qualitative and 8 studies included in the quantitative analysis. Most of the articles presented a low risk of bias. HBSS medium showed a superior ratio of cell viability compared to tap water (RR 0.26; 95% CI [0.21, 0.32]; p < 0.00001; I2 = 96%) and saline solution (RR 0.76; 95% CI [0.69, 0.84]; p < 0.0001; I2 = 99%). Herbal medicines showed a similar ratio of cell viability when compared to HBSS (RR 0.97; 95% CI [0.94, 1.00]; p = 0.08; I^2^ = 50%). Mixed results were observed between milk and HBSS: a superior ratio of HBSS was observed in an overall evaluation (RR 0.26; 95% CI [0.21, 0.32]; p < 0.00001; I^2^ = 96%), and a similar ratio was achieved when periodontal ligament (PDL) cells were removed prior to immersion in the solution (RR 0.94; 95% CI [0.87, 1.01]; p = 0.10; I2 = 0%) or rinsed in tap water or maintained in open air prior to immersion (RR 0.63; 95% CI [0.35, 1.12]; p = 0.11; I2 = not applicable). This systematic review and MA suggests that milk and herbal medicines could represent an alternative to HBSS. However, more studies are necessary to obtain a reliable conclusion.

## Introduction

Tooth avulsion is a traumatic injury characterized by the displacement of a tooth from its socket, resulting in the exposure of periodontal ligament cells and disruption of the blood supply to the pulp [1,2]. Immediate replantation of the teeth is indicated as the treatment of choice in many situations [3], providing healing to damaged tissue, with activation of clastic activity and rebuilding of periodontal fibers in damaged tissues [4,5].

However, in most cases, immediate replantation is impractical due to the circumstances of occurrence and distance to dentists [6]. Therefore, correct storage of the teeth is critical to preserving vital components, such as the periodontal ligament (PDL) cells and root cementum, as well as to reduce bacterial contamination [7].

The viability of PDL cells is critical to replantation of a tooth after avulsion, and the current evidence shows that this property can be affected by time, type of storage solution, and damage on the root surface [1,7].

The efficacy of different solutions in maintaining the viability of PDL cells has been tested. However, a valid answer about the effectiveness of such avulsed tooth storage solutions is still unavailable. Among the different solutions tested, Hank’s balanced salt solution (HBSS) has proven to be an active medium for preserving the viability, mitogenicity, and clonogenic capacity of periodontal ligament cells [8].

This systematic review and meta-analysis aimed to verify the capacity of different storage media to preserve the viability of PDL cells in comparison to HBSS.

## Material and methods

### Protocol and registration

This systematic review was registered at PROSPERO under the registration code CRD42016033187 and performed according to PRISMA (Preferred Reporting Items for Systematic Review and Meta-Analysis) guidelines (S1 PRISMA Checklist) [9].

### Search strategy and eligibility criteria

The PICO strategy was followed in this systematic review. Studies involving the periodontal ligament cells of human teeth from extraction or avulsion (P) stored in any experimental storage medium described in the literature (I) compared with that stored in HBSS (C), in which the primary outcome was the cell viability in this population (O). Opinion articles, technical articles, guides, and animal studies were excluded.

Searches were conducted in the following electronic databases, without language restriction, until December 2017: PubMed, Scopus, Web of Science, The Cochrane Library, and LILACS, followed by a grey literature search in OpenGrey and Google Scholar. All publications presented in the databases containing a combination of controlled pre-defined MeSH and free terms related to a storage medium for periodontal ligament cells used with Boolean operators (Or, And) to combine searches were retrieved. The previously defined terms were adapted to the rules of syntax of each bibliographic database (S1 Appendix).

After consultation of databases and the grey literature, all records were saved in a reference manager (EndNote, x7 version, Thomson Reuters, United States), and duplicated results were removed from the combination of the results obtained in all the surveyed sources. Additional citations were sought from the analysis of the reference list of all articles previously selected. The searches were conducted by two examiners (NCFF and LOB) and checked by a third examiner (RRL) in cases of disagreement.

After duplicate removal, the titles and abstracts that did not respect the established eligibility criteria were excluded. The resulting articles were evaluated and judged by their full texts.

### Data extraction

Data were extracted from the selected articles. A table was used to report the country, year of publication, study design, tooth origins, storage media, viability evaluation, period of storage, and results. This extraction was performed by two examiners (NCFF and LOB) and checked by a third examiner (RRL) in cases of divergence.

If necessary, in the case of absence of information that made data extraction or risk of bias evaluation unfeasible, attempts to contact the authors were made by e-mail. The contact attempt consisted of sending weekly e-mails for up to three consecutive weeks.

### Risk of bias

The selected studies were analyzed to determine the risk of bias, according to the Toxicological Data Reliability Assessment Tool (ToxRTool) [10], of which only the in vitro part was used. The instrument consists of 18 questions divided into five main criteria: Criteria Group I: test substance identification, test system characterization, study results documentation, plausibility of study design, and data. Each question could be answered as 1 (criterion met) or 0 (criterion not met). Additionally, the risk of bias was evaluated based on four central questions: "Is all information on the nature and physico-chemical properties of the test item given, which you deem indispensable for judging the data?"; "Is the number of replicates (or complete repetitions of the experiment) given?"; "Are the statistical methods for data analysis given and applied transparently?"; “Are the quantitative study results reliable?”.

At the end of the evaluation, each study was scored as 1 (reliable with no restrictions) for 15–18 final points; 2 (reliable with restrictions) for 11–14 final points; 3 (not reliable or not meeting all criteria) for a score of fewer than 11 final points; or 4 (not assignable) in cases of insufficient documentation for analysis. Studies with final scores of 1 or 2 were considered to have a low risk of bias. Studies with final scores of 3 or 4 were considered to have a high risk of bias. More detailed information about this tool and the criteria used in this review is available in S2 Appendix.

### Quantitative synthesis of the results

To carry out a quantitative synthesis of the results, meta-analyses were performed using the RevMan software (Review Manager v. 5.3, The Cochrane Collaboration; Copenhagen, Denmark). The meta-analyses were conducted to assess the relationship between periodontal ligament cell viability and the storage medium. Due to the different parameters analyzed in the studies and the various types of data provided in the results (continuous and dichotomous), five meta-analyses were performed.

Based on the principal comparisons of the included studies, five groups of storage medium were selected for subgroup analysis: milk, tap water, herbal medicines, saline solution, and HBSS (positive control). Thus, five meta-analyses were conducted: to compare dichotomous (1) data on cell viability when cultured cells were incubated in milk or HBSS; to examine continuous data (2) on cell viability when cultured cells were incubated in milk or HBSS; to compare cell viability when cultured cells were incubated in tap water versus HBSS (3); to compare cell viability when cultured cells were incubated in medicinal herbal medium versus HBSS (4); and to compare cell viability when cultured cells were incubated in saline solution versus HBSS (5). In all meta-analyses, the incubation time of periodontal ligament cells (PDL) considered was up to 1 h of storage in the medium. The 1-h period was chosen as default for this analysis due to the reported efficacy of HBSS during this time [7]. HBSS was used as a comparator in this systematic review.

Depending on the methodology of PDL cell culture, whenever possible, three subgroups were analyzed: removal of PDL cells prior immersion in the storage solution, immediate tooth immersion in the storage solution, and rinsing in tap water or open-air maintenance prior to immersion in the storage solution.

For dichotomous data (1st, 3rd, 4th, and 5th analysis), the prevalence of viable cells, expressed as a percentage of the total, in the primary storage medium was used to calculate the risk ratio (RR) with the 95% confidence interval (CI). For continuous data (second analysis) the average cell viability for each group (experimental and HBSS control) were used to calculate the mean difference with the 95% confidence interval (CI). A fixed-effects model was employed, because of the very small number of studies (five or fewer) included [11]. Heterogeneity was tested using the I^2^ index. In cases of high heterogeneity and whenever possible (meta-analysis in which no subgroup had three studies or more, or meta-analysis in which a subset had two studies or more per subgroup), a sensitivity analysis was performed [12].

If some of the information needed for the meta-analysis was absent from any of the selected studies, the authors were contacted to provide the missing data. Three attempts to contact the authors were made for each study. If, after the contact attempts, there was no response from the authors, the study was not included in the meta-analysis.

## Results

### Study selection and characteristics

A total of 693 records were identified from database searches, and 185 duplicate results were excluded. The 508 titles and abstracts found were evaluated according to the inclusion criteria, and 484 records were eliminated. The full texts of the remaining studies (n = 25) were assessed. Seven studies were excluded from this evaluation: one study was a review article [13], two studies were conducted on animal teeth [14,15], and four studies were excluded due to the absence of evaluation of cells from extracted or avulsed teeth [16–19].

**Fig 1:**
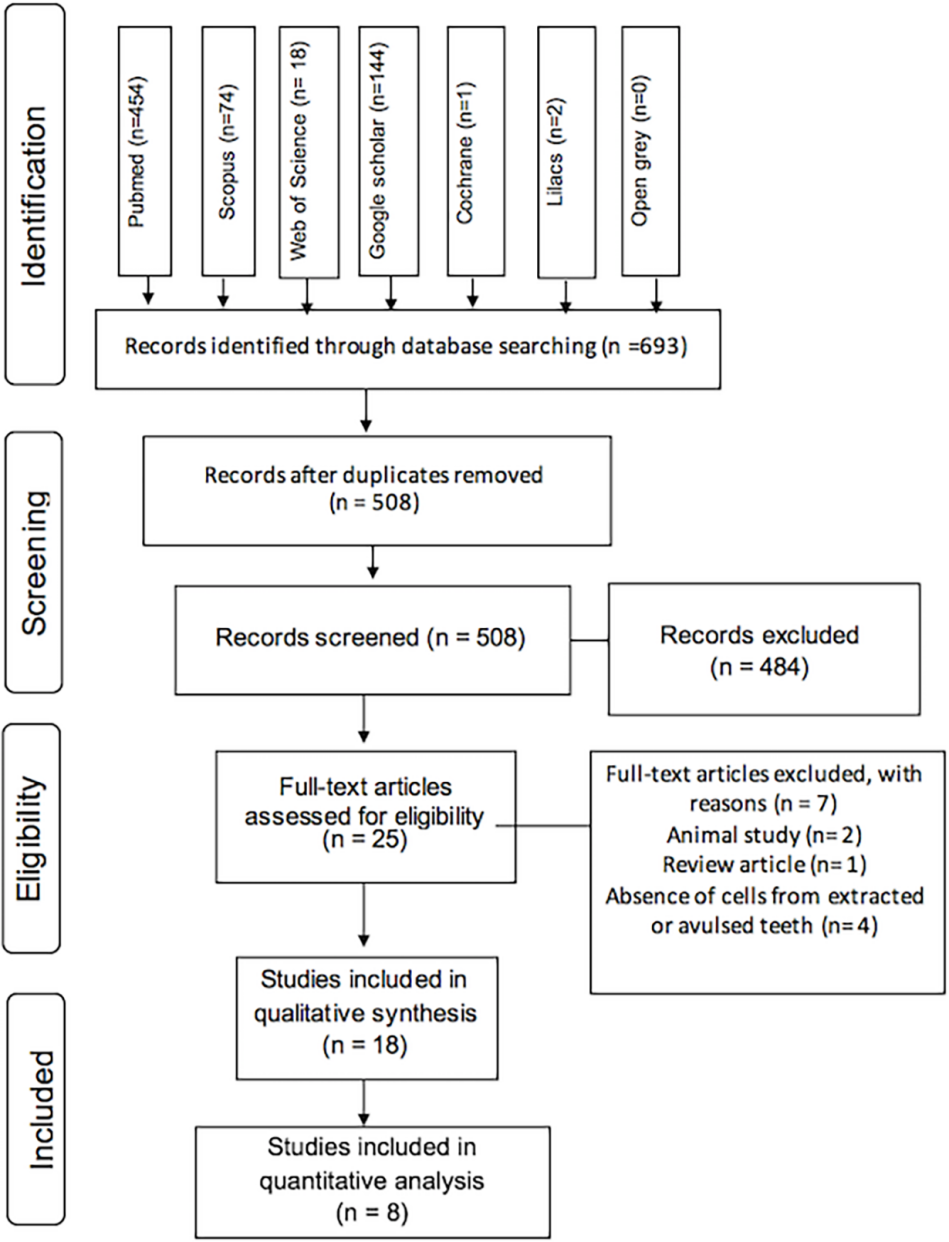
Flow diagram of literature search according to PRISMA statement.

A total of 18 articles [20–37] were selected and included in this systematic review. The selection process is summarized in Fig 1.

### Individual results of the included studies

Among the 18 studies evaluated, the performance of 34 solutions/substances were compared with that of HBSS regarding cellular viability. Bovine milk was used in 11 selected studies [20–22,25–27,29,30,33,35], tap water in seven studies [24,26–28,31,35,36], oral rehydration solution in three studies [29,31,32], and egg white in two studies [26,35]. Other solutions included phytotherapeutic solutions, such as propolis [24,35,36], green tea [33], Aloe vera [34,36], salvia [28], pomegranate [36], or even coconut water [37] and goat’s milk [35] (Table 1).

**Table 1:** Summary of characteristics of included studies.

	Studies	Teeth type /number	Storage media	Methodology	Cell viability evaluation method	Storage time	Main results
**A**	**Huang et al.,1996**[20]	Not specified/ Not informed	Whole milk, Alcon Opti- Free contact lens solution (sterile saline solution, 0.05% edetate disodium, and 0.001% polyquaternium), K-Mart contact lens solution (sterile saline solution, borate buffer, sorbic acid, and edetate disodium), and sterile saline. HBSS was a control group	Isolation of PDL cells prior incubation with the storage solutions	• Morphological Analysis • No statistical analysis.	0, 1, 3, 6, 10, 16, 24, 36, 48, 72, 96h.	The higher amount of attached cells was observed in the HBSS group after 72h of exposure.
**B**	**Olson et al.,1997**[21]	Healthy third molar and premolar/ Not informed	Whole milk, Gatorade, HBBS (save-a-tooth solution), HBBS+PDGF, Dry wells as negative control. EMEM as positive control,	Isolation of PDL cells prior incubation with the storage solutions.	• MTS/PMS reduction assay; • Two-way ANOVA complemented by the Student-Newman-Keuls test.	1, 2, 4, 8, and 12h.	The lowest cell viability was observed in the negative control, Gatorate and HBSS groups. HBSS with PDGF and milk presented cell viabilities similar to those of control group.
**C**	**Doyle et al., 1998**[22]	Non-impacted molars, premolars and anterior teeth free of periodontal disease/49	HBSS, milk, Negative control—2 h dry teeth. No dried teeth as positive control.	Dry teeth for 30, 60, and 90 min prior rehydration with storage media for 15 min.	• Trypan blue dye-exclusion assay; • ANOVA complemented by the Tukey-HSD post-hoc test	0.5, 1, 1.5h.	No significant difference in the number of viable cells at any of the dry storage times, with or without 15-min rehydration in HBSS or milk.
**D**	**Chung et al., 2004**[23]	Fully erupted healthy pre-molars/Not informed	F-medium, HBSS, and HBSS supplemented with 10, 100, and 500 nM chlorophyllin.	Isolation of PDL cells prior incubation with the storage solutions.	• MTT reduction assay; • Flow Cytometry- cell cycle; • ANOVA complemented by the Duncan´s test.	6 h	The highest cell viability was found in the PDL cells stored in HBSS supplemented with 500 nM chlorophyllin. No apoptosis, no necrosis detected in Flow cytometry.
**E**	**Ozan et al., 2007**[24]	Healthy third-molar teeth/Not informed	10% propolis solution, 20% propolis solution, long-shelf life light milk with lower fat content (milk), HBSS; tap water as negative control; DMEM as ositive control.	Isolation of PDL cells prior incubation with the storage solutions	• Trypan Blue dye-exclusion assay; • ANOVA complemented by the Tukey´s test.	1, 3, 6, 12, 24h.	The cell viabilities in the 10% propolis groups at 3, 6, 12, and 24 hours were significantly higher than those of HBSS and milk groups.
**F**	**Gopikrishnae et al., 2008**[25]	Human teeth with closed apices /55	• Coconut water, HBSS and milk; • Positive control: immediately treated with dispase and collagenase; • Negative control: dried for 8 hours, with no follow-up storage solution time, and then placed in the dispase and collagenase	Dry teeth for 30 min prior rehydration with storage media for 45 min. Recover the cells with dispase and collagenase	• Trypan Blue dye-exclusion assay; • Tukey-HSD multiple range test.	30 min	Coconut water group have presented a higher number of viable cells when compared to HBSS and milk.
**G**	**Khademi et al.,2008**[26]	Healthy premolars/100	• Milk and egg white; • Positive control: HBSS; • Negative control: tap water.	Teeth were immediately immersed into the storage media. Recover of the cells was done with trypsin and collagenase	• Trypan Blue dye-exclusion; • ANOVA complemented by the Tukey-HSD post-hoc test	1, 2, 4, 8, and 12h	There was no difference in the cell viability between egg white and HBSS media, but there was a statistically significant difference between the viability of PDL cells in egg white medium in comparison with milk and water.
**H**	**Ozan et al., 2008**[27]	Healthy third molar /Not informed	4.0%, 2.5%, 1.5%, and 0.5% of the juice of M. rubra fruit, Hank’s balanced salt solution (HBSS), phosphate-buffered saline (PBS), and tap water.	Isolation of PDL cells prior incubation with the storage solutions.	• Trypan Blue dye-exclusion assay; • ANOVA complemented by the Tukey´s test.	1, 3, 6, 12 or 24h	The cell viability of the 4.0% and 2.5% M. rubra groups were significantly higher than the other groups at 3, 6, and 12 hours and similar to HBSS at 24h.
**I**	**Ozan et al., 2008** [28]	Third molars/Not informed	S. officinalis solutions, Hank’s balanced salt solution (HBSS), phosphate buffered saline (PBS), and tap water	Isolation of PDL cells prior incubation with the storage solutions	• Trypan Blue dye-exclusion assay; • ANOVA complemented by the Tukey´s test.	1, 3, 6, 12, or 24h	The results showed 2.5% *S*. *officinalis* was a more effective storage medium than the other experimental solutions (p<0.05). Only at 1 hour and 3 hours was there found similar effect between 2.5% S. officinalis and HBSS. At 24 hours, 2.5% S. officinalis was found to be significantly better than the other solutions
**J**	**Rajendran et al.,2011**[29]	Healthy single-rooted premolars/30	• Ricetral, HBSS and milk; • Positive control: immediately assayed. • Negative control: dried for 8 hours, with no follow-up storage solution time, and then assayed.	Dry teeth for 30 min prior rehydration with storage media for 45 min. Recover the cells with collagenase and trypsin	• Trypan blue dye-exclusion assay; • Non parametric tests, Krusker–Wallis H-test, and Mann–Whitney U-test.	45 min	The positive control had the highest number of surviving cells, followed by HBSS, Ricetral, and milk groups in decreasing order.
**K**	**Esber et al., 2015**[30]	Single rooted healthy premolars/36	Probiotic yogurt, HBSS, saline and milk.	Dry teeth for 30 min prior rehydration with storage media for 45 min. Recover the cells with collagenase and dispase.	• Trypan blue dye-exclusion assay; • Non parametric ANOVA complemented by Kruskal-Wallis Test and Dunn’s Multiple Comparisons Test.	45 min	There was no significant difference in the number of viable PDL cells between HBSS, milk, Bifidibacterium animalis DN 173010 containing yogurt and saline. The teeth stored in positive control demonstrated the highest number of viable PDL cells.
**L**	**Jabarifar et al., 2015**[31]	Not informed	25%, 50%, and 100% Oral Rehydration Solution in comparison with HBSS) and tap water.	Isolation of PDL cells prior incubation with the storage solutions	• MTT reduction assay; • ANOVA and Post hoc (Duncan) tests.	2, 6, 12, 24, 48h	Oral Rehydration Solution preserved more viable cells and induced fewer apoptotic cells in comparison with HBSS
**M**	**Subramani-am et al., 2015**[32]	Premolars/130	HBSS; pasteurized, whole cold bovine milk and Oral Rehydration Solution-Liquid (ORS-L)	Dry teeth for 30 or 60 min prior rehydration with storage media for 45 or 90 min. Recover the cells with collagenase and dispase.	• Trypan Blue dye-exclusion assay; • One-way ANOVA complemented by the. Post hoc Tukey’s test.	45 and 90 min	ORS-L group presented similar cell viability as the HBSS group that was significantly higher than those of the milk group.
**N**	**Adeli et al., 2016**[33]	Impacted third molars/Not informed	DMEM, tap water, HBSS, whole milk, hypotonic sucrose solution (HSS), Green tea extract (GTE), and GTE+sucrose	Isolation of PDL cells prior incubation with the storage solutions	• MTT reduction assay; • ANOVA complemented by the Tukey´s test.	1, 2, 4 and 24h	All experimental groups presented similar cell viability values, except in HSS and tap water groups that had lower rate of viable cells in comparison to the others.
**O**	**Fulzele et al., 2016**[34]	Premolar /Not informed	HBSS, Aloevera gel and Packaged drinking water	Isolation of PDL cells prior incubation with the storage solutions.	• Trypan blue dye-exclusion assay; • ANOVA complemented by the Tukey´s test.	15, 30, 60, 90 and 120 min	DMEM, tap water, Hank's balanced salt solution (HBSS), whole milk, hypotonic sucrose solution, GTE, and GTE + sucrose No statistical significant difference was observed in cell viability between HBSS and *Aloevera* gel groups.
**P**	**Ulusoy et al., 2016**[35]	Healthy premolars/Not informed	Tap water, EMM, HBSS, UHT long-shelf-life lactose free cow milk, UHT long-shelf-life whole cow milk, UHT long-shelf-life skimmed cow milk, UHT long-shelf-life soy milk, UHT long-shelflife goat milk, UHT long shelf follow on milk with probiotic, 20% propolis, and egg white.	Isolation of PDL cells prior incubation with the storage solutions	• MTT reduction assay; • One-way ANOVA complemented by post hoc Duncan’s multiple comparison test.	1, 3, 6, 12, and 24h.	Goat milk displayed the highest capacity to maintain cell viability at all test intervals. Moreover, compared with all milks, HBSS performed significantly less effectively in maintaining PDL cell viability during the entire test period.
**Q**	**Babaji et al., 2017**[36]	Non specified/50	HBSS, propolis, Aloevera, and pomegranate juice	All the teeth were immersed immediately after extraction into respective storage media.	• Trypan Blue dye-exclusion assay; • ANOVA and multiple range using Tukey's test	45 min	Propolis showed more viable PDL cells followed by HBSS, *Alloevera*, and pomegranate.
**R**	**Saini et al., 2016**[37]	Premolars/69	HBSS, coconut milk, probiotic milk	All the teeth were rinsed under running tap water and left in open air for 20 min prior rehydration with storage media for 30 min. Recover the cells with collagenase and dispase.	• Trypan Blue dye-exclusion assay; • One-way ANOVA complemented by the. Post hoc Tukey’s test.	30 min	HBSS showed more viable PDL cells followed by Probiotic milk and Coconut milk. A comparison among media shown a highest capacity of HBSS to maintain PDL cells when compared to coconut milk and no difference between HBSS and probiotic milk.

In the studies evaluated, the substances that presented similar or superior performance in comparison with HBSS with respect to cellular viability of the PDL fibroblasts were coconut water [25], *Salvia officinallis* [28], goat milk [35], yogurt with probiotics [30], green tea [33], egg white [26,35], DMEM [24,33], Oral Rehydration Solution [29,31,32], HBSS supplemented with Chlorophyllin [23], HBSS augmented with PDGF [21], and Eagle's minimal essential medium (M-EM) [21,35]. The results using *Aloe vera* [34,36] and propolis [24,35,36] differed between studies, as shown in Table 1.

### Risk of bias

Among the evaluated studies, eight [24,27,30–32,34–36] were categorized as reliable with no restrictions, seven [21–23,28,29,33,37] as reliable with restrictions, and three studies [20,25,26] as not reliable (Table 2).

**Table 2:** Risk of bias evaluation according to ToxRTool.

Studies	A	B	C	D	E	F	G	H	I	J	K	L	M	N	O	P	Q	R
**Criteria Group I: Test substance identification**																		
Was the test substance identified?	1	1	1	1	1	1	1	1	1	1	1	1	1	1	1	1	1	1
Is the purity of the substance given?	0	1	1	1	1	0	0	1	0	0	0	0	1	1	0	0	1	1
Is information on the source/origin of the substance given?	0	0	0	1	1	0	1	0	1	0	1	1	1	1	1	1	1	1
Is all information on the nature and/or physico-chemical properties of the test item given, which you deem indispensable for judging the data?	1	1	1	1	1	0	0	0	0	0	1	1	1	1	1	0	1	0
**Criteria Group II: Test system characterization**																		
Is the test system described?	1	1	1	1	1	1	1	1	1	1	1	1	1	1	1	1	1	1
Is information given on the source/origin of the test system?	0	0	0	1	1	1	0	1	1	1	1	1	1	1	1	1	1	1
Are necessary information on test system properties, and on conditions of cultivation and maintenance given?	1	1	1	1	1	1	1	1	1	1	1	1	1	0	1	1	1	1
**Criteria Group III: Study design description**																		
Is the method of administration given?	1	1	1	1	1	1	0	1	1	1	0	1	1	1	1	1	1	1
Are doses administered or concentrations in application media given?	1	1	1	1	1	1	1	1	1	1	1	1	1	1	1	1	1	0
Are frequency and duration of exposure as well as time-points of observations explained?	1	1	1	1	1	0	1	1	1	1	1	1	1	1	1	1	0	1
Were negative controls included?	0	1	1	0	1	1	1	1	1	1	1	1	1	1	1	1	1	0
Were positive controls included?	0	1	1	0	1	1	1	1	1	1	1	1	1	1	1	1	1	1
Is the number of replicates (or complete repetitions of experiment) given?	0	0	0	0	0	0	0	0	1	0	1	0	0	0	0	1	1	1
**Criteria Group IV: Study results documentation**																		
Are the study endpoint(s) and their method(s) of determination clearly described?	1	1	1	1	1	0	1	1	1	1	1	1	1	1	1	1	1	1
Is the description of the study results for all endpoints investigated transparent and complete?	1	1	1	1	1	0	0	1	1	1	0	1	1	0	0	1	1	0
Are the statistical methods for data analysis given and applied in a transparent manner?	0	0	0	1	1	1	1	0	1	1	1	1	1	0	1	1	1	1
**Criteria Group V: Plausibility of study design and data**																		
Is the study design chosen appropriate for obtaining the substance-specific data aimed at?	0	1	1	1	1	1	1	0	1	1	1	1	1	1	1	1	1	1
Are the quantitative study results reliable?	0	0	0	0	1	1	0	1	1	1	1	1	1	0	1	1	1	1
Total	9	13	13	14	17	11	10	13	16	14	15	16	16	13	15	15	16	13
Category	3	2	2	2	1	3	3	2	1	2	1	1	1	2	1	1	1	2

Captions: A: Huang et al.,1996[20]; B: Olson et al.,1997[21]; C: Doyle et al., 1998[22]; D: Chung et al., 2004[23]; E: Ozan et al., 2007[24]; F: Gopikrishnae et al., 2008[25]; G: Khademi et al.,2008[26]; H: Ozan et al., 2008[27]; I: Ozan et al., 2008 [28]; J: Rajendran et al.,2011[29]; K: Esber et al., 2015[30]; L: Jabarifar et al., 2015[31]; M: Subramaniam et al., 2015[32]; N: Adeli et al., 2016[33]; O: Fulzele et al., 2016[34]; P: Ulusoy et al., 2016[35]; Q: Babaji et al., 2017[36]; R: Saini et al., 2016[37].

The risk of bias in studies was assessed based on four main questions. For the first question (“Is all information on the nature and/or physico-chemical properties of the test item given, which you deem indispensable for judging the data?”), seven [25–29,35,37] studies did not meet the criteria. For the second question [“Is the number of replicates (or complete repetitions of experiment) given?”], 13 studies [20–26,28,29,31–34] did not report these data. For the third question (“Are the statistical methods for data analysis given and applied in a transparent manner?”), five studies [20–22,28,33] did not meet the criteria. Finally, for the fourth question (“Are the quantitative study results reliable?”), six studies [20–23,33,34] did not present sufficient explanations of reliability. Considering the final score, 15 studies were classified as having a low risk of bias [21–24,27–37] and three as a high risk of bias [20,25,26] (Table 2).

### Quantitative analysis

Eight of the 18 studies included in this systematic review presented their results in percentage (%) of viable cells [20,22,24,26–28,31,34], eight studies presented in their results as the mean number of vital cells (continuous data) [21,25,29,30,32,35–37], and in two studies [23,33] the results were presented only in graphs. Only Rajendran et al. [29] responded to the e-mail contact but did not offer the requested data (percentage of viable cells in the analyzed groups). Due to the differences in presentation of data between the included articles and the absence of a response by e-mail contact, only studies that presented data in % or mean and standard deviation, for an incubation period of up to 1 h in the tested media, were included in the meta-analysis.

A high heterogeneity was found initially for all meta-analyses (1st: I^2^ = 98%, 2nd: I^2^ = 100%, 3rd: I^2^ = 96%, 4th I^2^ = 50%, and 5th: I^2^ = 99%). For this reason, a sensitivity analysis was performed. This process is described in Table 3.

**Table 3:** Sensitivity analysis process.

Meta-analysis	I^2^ range	Study excluded
1^st^ Milk (dichotomous data)	98 to 99%	None
2^nd^ Milk (continuous data)	99 to 100%	None
3^rd^ Tap water	95 to 98%	None
4^th^ Herbal medicines	50 to 84%	Fuzele et al. 2016
5^th^ Saline solution	-	-

**Fig 2:**
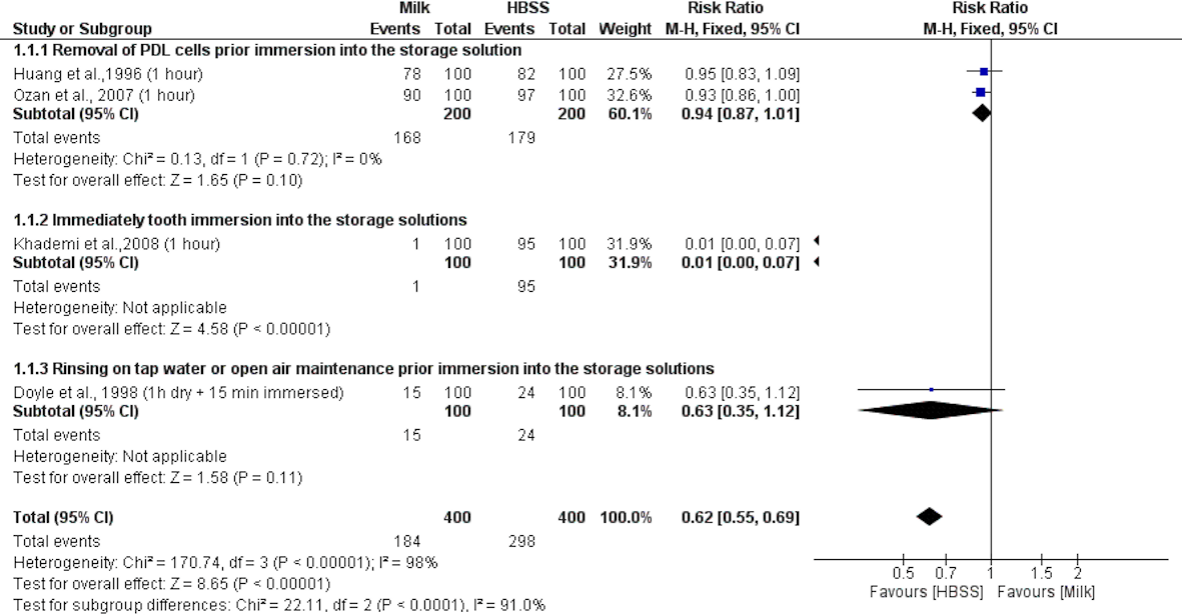
Forest plot of first meta-analysis: Cell viability of PDL cells (dichotomous data) incubated in milk or HBSS.

Four studies [20,22,24,26] were included in the 1st meta-analysis (Fig 2), comparing the viability of PDL cells stored in HBSS and milk. When PDL cells were removed prior to immersion in the storage solution or rinsed in tap water or maintained in open air prior immersion into the storage solutions, milk and HBSS presented similar results (RR 0.94 [0.87, 1.01]; p = 0.10; I^2^ = 0% and RR 0.63 [0.35, 1.12]; p = 0.11; I^2^ = not applicable, respectively). When the tooth was immediately immersed in the storage solution, HBSS presented higher PDL cell viability than milk (RR 0.01 [0.00, 0.07]; p < 0.0001; I^2^ = not applicable). In the overall analysis, HBSS medium showed a superior cell viability ratio to that of milk (RR 0.62 [0.55, 0.69]; p < 0.0001; I^2^ = 98%).

**Fig 3:**
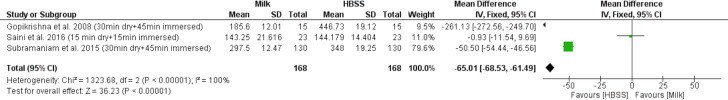
Forest plot of second meta-analysis: Cell viability of PDL cells (continuous data) incubated in milk or HBSS.

Three studies [25,32,37] were included in the 2nd meta-analysis (Fig 3) comparing continuous data on the viability of PDL cells stored in HBSS and milk. In all such studies, the tooth was either rinsed in tap water or maintained in open air prior to immersion in the storage solutions. HBSS medium showed a superior cell viability ratio to that of milk (RR -65.01 [-68.53, -61.49]; p < 0.0001; I^2^ = 100%).

**Fig 4:**
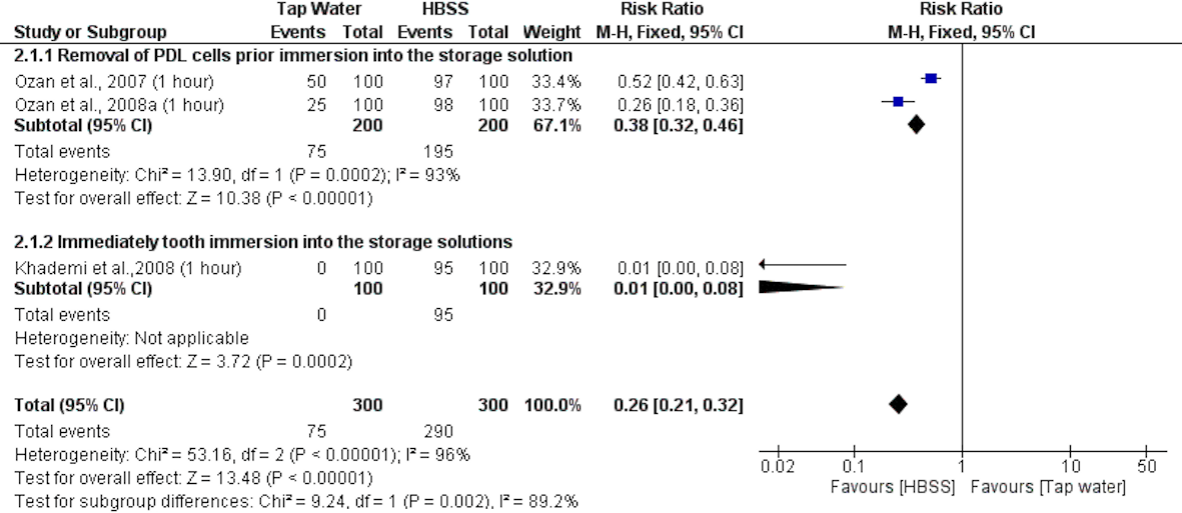
Forest plot of third meta-analysis: Cell viability of PDL cells incubated in tap water or HBSS.

Three studies [24,26,28] were included in the 3rd meta-analysis (Fig 4), comparing the viability of PDL cells stored in HBSS and tap water. Independently of the methodological manipulation of PDL cells, HBSS medium showed a superior ratio of cell viability to that of tap water (overall RR 0.26 [0.21, 0.32]; p < 0.00001; I^2^ = 96%).

**Fig 5:**
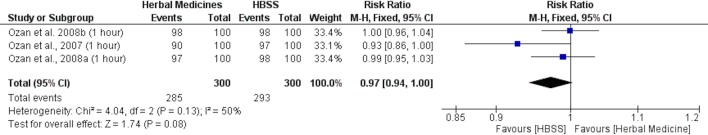
Forest plot of third meta-analysis: Cell viability of PDL cells (continuous data) incubated in medicinal herbal medium versus HBSS.

Three studies [24,27,28] were included in the 4th meta-analysis (Fig 5), comparing the viability of PDL cells stored in HBSS and herbal medicines. In all such studies, the PDL cells were removed prior to immersion in the storage medium. As storage media, HBSS and herbal medicines showed similar cell viability ratios (RR 0.97 [0.94, 1.00]; p = 0.08; I2 = 50%).

**Fig 6:**
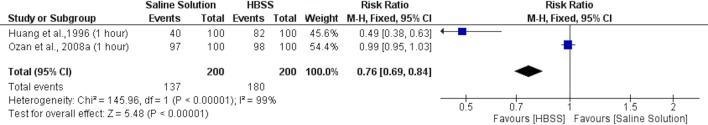
Forest plot of third meta-analysis: Cell viability of PDL cells (continuous data) incubated in saline solution versus HBSS.

Two studies [20,28] were included in the 5th meta-analysis (Fig 6), comparing the viability of PDL cells stored in HBSS and saline solution. For this meta-analysis, duplicated data [27,28] were considered only once; therefore, data from Ozan et al. [28] were not included. In both studies, the PDL cells were removed prior to immersion in the storage medium. HBSS showed a superior cell viability ratio to that of saline solution (RR 0.76 [0.69, 0.84]; p < 0.0001; I^2^ = 99%).

## Discussion

Concerned with the effectiveness of storage medium for maintaining PDL cell viability in avulsed teeth prior to replantation, this systematic review retrieved data on 34 different solutions used for this purpose. HBSS was considered the gold standard for this study. A qualitative evaluation showed that the capacity of HBSS to maintain PDL cell viability was similar or superior to that of coconut water [25], *Salvia officinallis* [28], goat milk [35], yogurt with probiotics [30], green tea [33], egg white [26,35], DMEM [24,33], Oral Rehydration Solution [29,31,32], HBSS supplemented with Chlorophyllin [23], HBSS supplemented with PDGF [21] and Eagle's minimal essential medium (M-EM) [21,35]. On meta-analysis evaluation, HBSS presented a higher level of viable PDL cells in comparison to tap water and saline solutions, and a similar level when compared to herbal medicines.

PDL cells are responsible for maintaining the overall integrity of the periodontal ligament, contributing to homeostasis and repair of damaged tissue [38,39]. In cases of avulsion, transplantation, or intentional extraction of the tooth, it is suggested that the maintenance of PDL cell viability is critical to preventing ankylosis and resorption, in cases of tooth replantation [40].

In most clinical situations, immediate tooth replantation is not suitable, and the avulsed tooth must be stored [1,3]. During this storage time, it is important to ensure the maintenance of viability, differentiation, and proliferative capacities of the periodontal ligament cells, along with the preservation of the cemented layer. Among these aspects, this systematic review and meta-analysis focused on the evaluation of the effectiveness of different storage media for maintaining the viability of periodontal ligament cells in comparison to HBSS.

Systematic reviews are subsequent studies that can summarize clinical questions based on an evaluation of published studies through a solid method [12,41]. In this type of study, a quantitative assessment of the problem is possible through a meta-analysis, which compares data from each study in a statistical analysis weighted according to heterogeneity among studies [11]. Thus, this systematic review could help to clarify the evidence in this field, improving the information available to clinicians and society about how to proceed in the clinical situation of tooth avulsion.

Among the selected studies, the process of evaluation of PDL cells followed these patterns: ten studies isolated PDL cells prior to incubation with the storage solutions [20,21,23,24,27,28,31,33–35], and in the remaining eight studies, the PDL cells were removed after incubation [22,25,26,29,30,32,36,37]. Isolating PDL cells prior to storage in the selected solution may be the nearest approximation of a clinical condition of avulsion or tooth transplantation.

In comparison to HBSS, 34 solutions were used among the 18 studies included (20–37), with a time of immersion varying from 15 min to 96 h. Comparison between the performance of the solutions in the different studies presents some obstacles due to this considerable variability in time. It is known that temperature and duration of exposure are factors affecting the performance of storage media [7].

Among most of the included articles, the incubation temperature in storage medium varied from 20 to 37°C [21,23,24,26–28,31,33–35,37]. Only one study compared the effect of temperature on cell viability [20], showing better maintenance of viability in milk at room temperature when compared to lower temperatures. Thus, it is considered safe to manipulate the solutions tested in these studies at room temperature.

Whole bovine milk was the most frequently evaluated storage medium applied to maintain the viability of periodontal ligament cells, and it was tested in 11 studies [20–22,24–26,29,30,33,35], showing contrasting results. Nine [20,24–26,29,30,33,35] of these 11 studies indicated that milk presented a worse outcome than HBSS. The possible properties responsible for the maintenance of periodontal ligament cell viability were primarily related to its osmolarity, adequate pH, and presence of nutrients [4,13]. Other forms of milk, such as skimmed milk [35] and lactose-free milk [35], have the capacity to maintain the viability of the tested PDL cells but also presented worse results than HBSS.

Different herbal/vegetal extracts were tested in the included studies, such as propolis [24,35,36], Salvia officinalis [28], Morus rubra fruit [27], Aloe vera [34,36], green tea [33] and pomegranate [36]. Except for propolis, which was evaluated by three studies at different concentrations and presented different results [24,35,36], the other solutions presented higher viability of PDL cells than HBSS [27,28,33,34,36]. The contrasting results presented by propolis could be related to the main characteristics of the solutions in the studies. In one study, which showed better viability of PDL cells than HBSS, a DMEM solution was added to propolis extract [24]. In the other two studies, in which the behavior was worse than that of HBSS, the propolis extract was dissolved in ethanol [35,36].

The incomplete reporting of the sample characteristics may represent a pitfall of the included studies. Among the 18 studies, only 9 studies [22,25,26,29,30,32,36,37] specified the number of human teeth used, which varied from 30 to 130 teeth. Regarding the type of teeth used, most of the studies specified the type [21–24,26–30,33–37], which varied from premolars to third molars. However, it is suggested that the type of tooth employed in the study did not result in significant differences in PDL cells [42].

The evaluation of PDL cell viability was performed using the Trypan blue dye-exclusion assay in 12 studies [22,24–30,32,34,36,37], 4 studies [23,31,33,35] used the 3-(4,5-Dimethylthiazol-2-yl)-2,5-diphenyltetrazolium bromide (MTT) reduction assay, 1 study used the tetrazolium compound [3-(4,5-dimethylthiazol-2-yl)-5-(3-carboxymethoxyphenyl)-2-(4-sulfophenyl)-2H-tetrazolium, inner salt] (MTS) and an electron coupling reagent phenazine methosulphate (PMS), briefly called MTS/PMS reduction assay [21], and 1 study [20] evaluated PDL cells only by morphologic analysis. One must keep in mind that the Trypan blue exclusion assay is the only one that assess the actual number of viable cells, whereas the other methods only evaluate mitochondrial cell metabolism [43,44].

To evaluate the methodological quality of the selected studies, a checklist focused on evaluating the reliability of toxicological data was built [10]. Three studies were classified as having a high risk of bias [20,25,26], which may compromise the presented results. The observed methodological flaws in the included studies were concentrated on the absence of chemical property information [25–29,35,37], lack of information about replicates [20–26,28,29,31–34], poor description of statistical tests [20–22,28,33], and inadequate data replication [20–23,26,34].

The quantitative evaluation was conducted through five meta-analyses. Among these, four substances were compared with HBSS: milk, tap water, saline solutions, and herbal medicines. Milk and tap water presented lower levels of viable PDL cells than HBSS. In most studies, these two substances were used as negative controls due to their inability to maintain pH or adequate nutrients [7,45].

The period of storage used in the meta-analyses was up to 1 h. Due to the substantial variation in the period of storage (15 min to 96 h) among studies [20–37], the selected period seems to be a closer approximation to clinical practice. Overall, the tested substances presented an indirect association between the rates of viability and the exposure time [20–37].

In the quantitative analysis, milk was evaluated in two different meta-analyses. In the overall analysis, HBSS presented the best maintenance of PDL cell viability, which conforms with the results of most of the studies included in this systematic review [20,24–26,29,30,33,35]. However, HBSS presented a similar ratio when the PDL cells were removed prior immersion in the storage solution or rinsed in tap water or maintained in open air prior to immersion in the storage solutions, milk and HBSS presented similar results. These results may be related to the properties of milk and its sensitivity to time and temperature [46,47]. Moreover, milk has been associated with a high susceptibility to contamination and an inability to retain lost cellular metabolites [13,30].

The herbal medicines, when evaluated as a whole, presented similar viability ratios to HBSS. In the included studies, all herbal medicines were associated with antioxidant properties [24,27,28]. The main reason pointed out by study authors for the use of these substances was their potential antioxidant and anti-inflammatory properties. However, a lack of evidence was observed associated with the main properties of herbal medicines in dental tissues.

Even though some chemical information on commercial products in many storage media used is unnecessary, critical details of the composition of plant extracts or original experimental solutions are essential to evaluate their effect on the viability of PDL cells [7,48]. Moreover, although the flaws in data evaluation and analysis observed in the studies included did not compromise the methodological quality as a whole, these problems may induce bias on data analysis [49,50].

The main limitation of this study was the high heterogeneity observed through meta-analysis by I^2^ index. Four meta-analyses (Figs 2, 3, 4 and 6) presented high heterogeneity values, varying from 96% to 100%. This result indicates the high variability among the included studies, which can compromise the reliability of the conclusion drawn from a meta-analysis [11]. Indeed, more homogeneous studies, with a randomized sample and comparable process of PDL cell evaluation could help to clarify this question.

## Conclusion

This systematic review and meta-analysis suggests that some solutions, such as milk and herbal medicine, could represent an alternative means of maintaining the viability of PDL cells prior to replantation of the tooth. Even though additional studies related to the maintenance of PDL cells are still necessary, the current evidence suggests that some solutions could be useful to maintain PDL cell viability.

## Supporting information

S1 PRISMA Checklist (DOC)

S1 Appendix Terms used on database search. (DOCX)

S2 Appendix Criteria considered in risk of bias evaluation according to ToxRTool. (DOCX)

## References

[1] Martins-Júnior PA, Franco FA da S, de Barcelos RV, Marques LS, Ramos-Jorge ML. Replantation of avulsed primary teeth: a systematic review. Int J Paediatr Dent [Internet]. 2014 3;24(2):77–83. Available from: http://www.ncbi.nlm.nih.gov/pubmed/24205851 10.1111/ipd.12075 24205851

[2] Pohl Y, Filippi A, Kirschner H. Results after replantation of avulsed permanent teeth. I. Endodontic considerations. Dent Traumatol [Internet]. 2005 4 [cited 2017 Sep 28];21(2):80–92. Available from: http://www.ncbi.nlm.nih.gov/pubmed/15773887 10.1111/j.1600-9657.2004.00297.x 15773887

[3] Andersson L, Andreasen JO, Day P, Heithersay G, Trope M, DiAngelis AJ, et al Guidelines for the Management of Traumatic Dental Injuries: 2. Avulsion of Permanent Teeth. Pediatr Dent [Internet]. 2016 10 [cited 2017 Sep 29];38(6):369–76. Available from: http://www.ncbi.nlm.nih.gov/pubmed/27931479 27931479

[4] Day P, Duggal M. Interventions for treating traumatised permanent front teeth: avulsed (knocked out) and replanted In: Day P, editor. Cochrane Database of Systematic Reviews [Internet]. Chichester, UK: John Wiley & Sons, Ltd; 2010 [cited 2017 Sep 29]. p. CD006542. Available from: http://www.ncbi.nlm.nih.gov/pubmed/20091594 10.1002/14651858.CD006542.pub2 20091594

[5] Pohl Y, Filippi A, Kirschner H. Results after replantation of avulsed permanent teeth. II. Periodontal healing and the role of physiologic storage and antiresorptive-regenerative therapy. Dent Traumatol [Internet]. 2005 4;21(2):93–101. Available from: http://www.ncbi.nlm.nih.gov/pubmed/15773888 10.1111/j.1600-9657.2004.00298.x 15773888

[6] Andreasen JO, Lauridsen E, Gerds TA, Ahrensburg SS. Dental Trauma Guide: A source of evidence-based treatment guidelines for dental trauma. Dent Traumatol [Internet]. 2012 10 [cited 2017 Sep 29];28(5):345–50. Available from: http://www.ncbi.nlm.nih.gov/pubmed/22994505 10.1111/j.1600-9657.2011.01059_1.x 22994505

[7] Khinda VI, Kaur G, Brar GS, Kallar S, Khurana H. Clinical and Practical Implications of Storage Media used for Tooth Avulsion. Marwah N, editor. Int J Clin Pediatr Dent [Internet]. 2017 [cited 2017 Sep 28];10(2):158–65. Available from: http://www.ncbi.nlm.nih.gov/pubmed/28890616 10.5005/jp-journals-10005-1427 28890616 PMC5571385

[8] Ashkenazi M, Marouni M, Sarnat H. In vitro viability, mitogenicity and clonogenic capacities of periodontal ligament fibroblasts after storage in four media supplemented with growth factors. Dent Traumatol [Internet]. 2001 2;17(1):27–35. Available from: http://www.ncbi.nlm.nih.gov/pubmed/11475768 11475768 10.1034/j.1600-9657.2001.170106.x

[9] Moher D, Liberati A, Tetzlaff J, Altman DG. Preferred reporting items for systematic reviews and meta-analyses: the PRISMA statement. Ann Intern Med [Internet]. 2009 8 18 [cited 2015 Sep 2];151(4):264–9, W64. Available from: http://www.ncbi.nlm.nih.gov/pubmed/19622511 19622511 10.7326/0003-4819-151-4-200908180-00135

[10] Schneider K, Schwarz M, Burkholder I, Kopp-Schneider A, Edler L, Kinsner-Ovaskainen A, et al “ToxRTool”, a new tool to assess the reliability of toxicological data. Toxicol Lett [Internet]. 2009 9 10 [cited 2017 May 3];189(2):138–44. Available from: http://www.ncbi.nlm.nih.gov/pubmed/19477248 10.1016/j.toxlet.2009.05.013 19477248

[11] Borenstein M, Hedges L V., Higgins JPT, Rothstein HR. Introduction to Meta-Analysis [Internet]. Chichester, UK: John Wiley & Sons, Ltd; 2009 Available from: http://doi.wiley.com/10.1002/9780470743386

[12] Higgins JPT, Green S, editors. Cochrane handbook for systematic reviews of interventions version 5.1.0 [updated March 2011] [Internet]. The Cochrane Collaboration; 2011 Available from: http://handbook.cochrane.org

[13] Krasner PR. Treatment of tooth avulsion in the emergency department: Appropriate storage and transport media. Am J Emerg Med [Internet]. 1990;8(4):351–5. Available from: http://www.sciencedirect.com/science/article/pii/073567579090095H 2194470 10.1016/0735-6757(90)90095-h

[14] Hupp JG, Mesaros S V, Aukhil I, Trope M. Periodontal ligament vitality and histologic healing of teeth stored for extended periods before transplantation. Endod Dent Traumatol. 1998/04/29. 1998;14(2):79–83. 9558520 10.1111/j.1600-9657.1998.tb00815.x

[15] Casaroto AR, Hidalgo MM, Sell AM, Franco SL, Cuman RK, Moreschi E, et al Study of the effectiveness of propolis extract as a storage medium for avulsed teeth. Dent Traumatol. 2010/07/29. 2010;26(4):323–31. 10.1111/j.1600-9657.2010.00879.x 20662885

[16] Blomlof L, Otteskog P, Hammarstrom L. Effect of storage in media with different ion strengths and osmolalities on human periodontal ligament cells. Scand J Dent Res. 1981/04/01. 1981;89(2):180–7. 6943665 10.1111/j.1600-0722.1981.tb01669.x

[17] Hwang JY, Choi SC, Park J-H, Kang SW. The Use of Green Tea Extract as a Storage Medium for the Avulsed Tooth. J Endod [Internet]. 2011;37(7):962–7. Available from: http://www.sciencedirect.com/science/article/pii/S0099239911004195 10.1016/j.joen.2011.03.028 21689552

[18] Zhan X, Zhang C, Dissanayaka WL, Cheung GS, Jin L, Yang Y, et al Storage media enhance osteoclastogenic potential of human periodontal ligament cells via RANKL-independent signaling. Dent Traumatol. 2012/04/11. 2013;29(1):59–65. 10.1111/j.1600-9657.2012.01138.x 22487464

[19] Souza BD, Luckemeyer DD, Reyes-Carmona JF, Felippe WT, Simoes CM, Felippe MC. Viability of human periodontal ligament fibroblasts in milk, Hank’s balanced salt solution and coconut water as storage media. Int Endod J. 2010/11/19. 2011;44(2):111–5. 10.1111/j.1365-2591.2010.01809.x 21083571

[20] Huang SC, Remeikis NA, Daniel JC. Effects of long-term exposure of human periodontal ligament cells to milk and other solutions. J Endod [Internet]. 1996 1;22(1):30–3. Available from: http://www.ncbi.nlm.nih.gov/pubmed/8618083 10.1016/S0099-2399(96)80233-0 8618083

[21] Olson BD, Mailhot JM, Anderson RW, Schuster GS, Weller RN. Comparison of various transport media on human periodontal ligament cell viability. J Endod. 1997;23(11):676–9. 10.1016/S0099-2399(97)80399-8 9587306

[22] Doyle DL, Dumsha TC, Sydiskis RJ. Effect of soaking in Hank’s balanced salt solution or milk on PDL cell viability of dry stored human teeth. Endod Dent Traumatol [Internet]. 1998 10;14(5):221–4. Available from: http://www.ncbi.nlm.nih.gov/pubmed/9855801 9855801 10.1111/j.1600-9657.1998.tb00843.x

[23] Chung W-G, Lee EJ, Lee S-J, Lee S-A, Kim J. Effect of chlorophyllin on normothermic storage of human periodontal ligament cells. J Endod [Internet]. 2004 6;30(6):399–402. Available from: http://www.ncbi.nlm.nih.gov/pubmed/15167465 15167465 10.1097/00004770-200406000-00005

[24] Ozan F, Polat ZA, Er K, Ozan U, Değer O. Effect of propolis on survival of periodontal ligament cells: new storage media for avulsed teeth. J Endod [Internet]. 2007 5;33(5):570–3. Available from: http://www.ncbi.nlm.nih.gov/pubmed/17437874 10.1016/j.joen.2006.12.021 17437874

[25] Gopikrishna V, Thomas T, Kandaswamy D. A quantitative analysis of coconut water: a new storage media for avulsed teeth. Oral Surg Oral Med Oral Pathol Oral Radiol Endod [Internet]. 2008 2;105(2):e61–5. Available from: http://www.ncbi.nlm.nih.gov/pubmed/18230380 10.1016/j.tripleo.2007.08.003 18230380

[26] Khademi AA, Saei S, Mohajeri MR, Mirkheshti N, Ghassami F, Torabi nia N, et al A new storage medium for an avulsed tooth. J Contemp Dent Pract [Internet]. 2008 9 1;9(6):25–32. Available from: http://www.ncbi.nlm.nih.gov/pubmed/18784856 18784856

[27] Ozan F, Tepe B, Polat ZA, Er K. Evaluation of in vitro effect of Morus rubra (red mulberry) on survival of periodontal ligament cells. Oral Surg Oral Med Oral Pathol Oral Radiol Endod [Internet]. 2008 2;105(2):e66–9. Available from: http://www.ncbi.nlm.nih.gov/pubmed/18230381 10.1016/j.tripleo.2007.08.002 18230381

[28] Ozan F, Polat ZA, Tepe B, Er K. Influence of storage media containing Salvia officinalis on survival of periodontal ligament cells. J Contemp Dent Pract [Internet]. 2008 9 1;9(6):17–24. Available from: http://www.ncbi.nlm.nih.gov/pubmed/18784855 18784855

[29] Rajendran P, Varghese NO, Varughese JM, Murugaian E. Evaluation, using extracted human teeth, of Ricetral as a storage medium for avulsions—an in vitro study. Dent Traumatol [Internet]. 2011 6;27(3):217–20. Available from: http://www.ncbi.nlm.nih.gov/pubmed/21535405 10.1111/j.1600-9657.2011.00988.x 21535405

[30] Esber C, Peker S, Durhan MA, Kulan P, Kuscu OO, Pisiriciler R, et al A Quantitative Analysis of a Probiotic Storage Media for Avulsed Teeth. Acta Stomatol Croat [Internet]. 2015 3;49(1):21–6. Available from: http://www.ncbi.nlm.nih.gov/pubmed/27688382 10.15644/asc49/1/3 27688382 PMC4945343

[31] Jabarifar SE, Razavi SM, Haje Norouzali Tehrani M, Roayaei Ardekani M. The effect of Oral Rehydration Solution on apoptosis of periodontal ligament cells. Dent Traumatol [Internet]. 2015 8;31(4):283–7. Available from: http://www.ncbi.nlm.nih.gov/pubmed/25865050 10.1111/edt.12173 25865050

[32] Subramaniam P, Girija P, Eswara U, Girish Babu KL. Oral rehydration salt-liquid as a storage medium for avulsed tooth. Dent Traumatol [Internet]. 2015 2;31(1):62–6. Available from: http://www.ncbi.nlm.nih.gov/pubmed/25263952 10.1111/edt.12127 25263952

[33] Adeli F, Zabihi E, Abedian Z, Gharekhani S, Pouramir M, Khafri S, et al Comparative in vitro study of the effectiveness of Green tea extract and common storage media on periodontal ligament fibroblast viability. Eur J Dent [Internet]. 2016;10(3):408–12. Available from: http://www.ncbi.nlm.nih.gov/pubmed/27403063 10.4103/1305-7456.184158 27403063 PMC4926598

[34] Fulzele P, Baliga S, Thosar N, Pradhan D. Evaluation of Aloevera Gel as a Storage Medium in Maintaining the Viability of Periodontal Ligament Cells—An in Vitro Study. J Clin Pediatr Dent [Internet]. 2016;40(1):49–52. Available from: http://www.ncbi.nlm.nih.gov/pubmed/26696107 10.17796/1053-4628-40.1.49 26696107

[35] Ulusoy AT, Kalyoncuoglu E, Kaya S, Cehreli ZC. Evaluation of goat milk as storage media to preserve viability of human periodontal ligament cells in vitro. Dent Traumatol [Internet]. 2016 8;32(4):264–8. Available from: http://www.ncbi.nlm.nih.gov/pubmed/26635107 10.1111/edt.12245 26635107

[36] Babaji P, Melkundi M, Devanna R, Suresh B, Chaurasia V, Gopinath P. In vitro comparative evaluation of different storage media (hank’s balanced salt solution, propolis, Aloe vera, and pomegranate juice) for preservation of avulsed tooth. Eur J Dent [Internet]. 2017 [cited 2017 Apr 20];11(1):71 Available from: http://www.eurjdent.com/text.asp?2017/11/1/71/202612 10.4103/ejd.ejd_101_16 28435369 PMC5379839

[37] Saini D, Gadicherla P, Chandra P, Anandakrishna L. Coconut milk and probiotic milk as storage media to maintain periodontal ligament cell viability: an in vitro study. Dent Traumatol [Internet]. 2017 6 [cited 2017 Sep 28];33(3):160–4. Available from: http://www.ncbi.nlm.nih.gov/pubmed/27943593 10.1111/edt.12310 27943593

[38] Hasegawa T, Chosa N, Asakawa T, Yoshimura Y, Ishisaki A, Tanaka M. Establishment of immortalized human periodontal ligament cells derived from deciduous teeth. Int J Mol Med [Internet]. 2010 11;26(5):701–5. Available from: http://www.ncbi.nlm.nih.gov/pubmed/20878092 20878092 10.3892/ijmm_00000516

[39] Agarwal S, Chandra CS, Piesco NP, Langkamp HH, Bowen L, Baran C. Regulation of periodontal ligament cell functions by interleukin-1beta. Infect Immun [Internet]. 1998 3;66(3):932–7. Available from: http://www.ncbi.nlm.nih.gov/pubmed/9488378 9488378 10.1128/iai.66.3.932-937.1998 PMC107998

[40] Tuna EB, Arai K, Tekkesin MS, Seymen F, Gencay K, Kuboyama N, et al Effect of fibroblast growth factor and enamel matrix derivative treatment on root resorption after delayed replantation. Dent Traumatol [Internet]. 2015 2 [cited 2017 Sep 28];31(1):49–56. Available from: http://www.ncbi.nlm.nih.gov/pubmed/25290558 10.1111/edt.12141 25290558

[41] Liberati A, Altman DG, Tetzlaff J, Mulrow C, Gøtzsche PC, Ioannidis JPA, et al The PRISMA Statement for Reporting Systematic Reviews and Meta-Analyses of Studies That Evaluate Health Care Interventions: Explanation and Elaboration. PLoS Med [Internet]. 2009 7 21 [cited 2017 Mar 28];6(7):e1000100 Available from: http://www.ncbi.nlm.nih.gov/pubmed/19621070 10.1371/journal.pmed.1000100 19621070 PMC2707010

[42] Marchesan JT, Scanlon CS, Soehren S, Matsuo M, Kapila YL. Implications of cultured periodontal ligament cells for the clinical and experimental setting: a review. Arch Oral Biol [Internet]. 2011 10 [cited 2017 Sep 28];56(10):933–43. Available from: http://www.ncbi.nlm.nih.gov/pubmed/21470594 10.1016/j.archoralbio.2011.03.003 21470594 PMC3132241

[43] Stoddart MJ. Cell Viability Assays: Introduction. In 2011. p. 1–6. Available from: http://link.springer.com/10.1007/978-1-61779-108-6_1 10.1007/978-1-61779-108-6_1 21468961

[44] Strober W. Trypan Blue Exclusion Test of Cell Viability In: Current Protocols in Immunology [Internet]. Hoboken, NJ, USA: John Wiley & Sons, Inc.; 1964 Available from: http://doi.wiley.com/10.1002/0471142735.ima03bs21

[45] Bağ İ, Yildirim S. Effect of avulsion storage media on periodontal ligament fibroblast differentiation. Dent Traumatol [Internet]. 2017 8 31 [cited 2017 Sep 28]; Available from: http://www.ncbi.nlm.nih.gov/pubmed/28715096 10.1111/edt.12356 28715096

[46] Day PF, Gregg TA, Ashley P, Welbury RR, Cole BO, High AS, et al Periodontal healing following avulsion and replantation of teeth: a multi-centre randomized controlled trial to compare two root canal medicaments. Dent Traumatol [Internet]. 2012 2 [cited 2017 Sep 29];28(1):55–64. Available from: http://www.ncbi.nlm.nih.gov/pubmed/21988960 10.1111/j.1600-9657.2011.01053.x 21988960

[47] Krasner P, Person P. Preserving avulsed teeth for replantation. J Am Dent Assoc [Internet]. 1992 11 [cited 2017 Oct 3];123(11):80–8. Available from: http://www.ncbi.nlm.nih.gov/pubmed/1469209 1469209 10.14219/jada.archive.1992.0300

[48] Er K, Polat ZA, Özan F, Taşdemir T, Sezer U, Siso ŞH. Cytotoxicity Analysis of Strontium Ranelate on Cultured Human Periodontal Ligament Fibroblasts: A Preliminary Report. J Formos Med Assoc [Internet]. 2008 8 [cited 2017 Sep 28];107(8):609–15. Available from: http://www.ncbi.nlm.nih.gov/pubmed/18678544 10.1016/S0929-6646(08)60178-3 18678544

[49] Higgins JPT, Altman DG, Gøtzsche PC, Jüni P, Moher D, Oxman AD, et al The Cochrane Collaboration’s tool for assessing risk of bias in randomised trials. BMJ [Internet]. 2011 10 18 [cited 2017 Jan 29];343:d5928 Available from: http://www.ncbi.nlm.nih.gov/pubmed/22008217 10.1136/bmj.d5928 22008217 PMC3196245

[50] Saltaji H, Ospina MB, Armijo-Olivo S, Agarwal S, Cummings GG, Amin M, et al Evaluation of risk of bias assessment of trials in systematic reviews of oral health interventions, 1991–2014. J Am Dent Assoc [Internet]. 2016 Sep [cited 2017 Oct 16];147(9):720–728.e1. Available from: http://www.ncbi.nlm.nih.gov/pubmed/27155754. 10.1016/j.adaj.2016.03.017 27155754

