# Correction: Efficacy of Hank's balanced salt solution compared to other solutions in the preservation of the periodontal ligament. A systematic review and meta-analysis

**DOI:** 10.1371/journal.pone.0209867

**Published:** 2018-12-20

**Authors:** Nathalia Carolina Fernandes Fagundes, Leonardo Oliveira Bittencourt, Marcela Baraúna Magno, Márcia Martins Marques, Lucianne Cople Maia, Rafael Rodrigues Lima

The affiliation of the fifth author is incorrect. Lucianne Cople Maia is not affiliated with #2 but with #3 Department of Pediatric Dentistry and Orthodontics, School of Dentistry, Universidade Federal do Rio de Janeiro, Rio de Janeiro, Brazil.
