# Supplementary material for: Efficacy of Hank's balanced salt solution compared to other solutions in the preservation of the periodontal ligament. A systematic review and meta-analysis
Source: PLoS One. 2018 Jul 13;13(7):e0200467. doi: 10.1371/journal.pone.0200467 (PMC6044542; doi:10.1371/journal.pone.0200467)
Supplement: S1 Appendix — (DOCX) [file pone.0200467.s002.docx]

S1 Appendix: Terms used on database search.

| Database | Search format |
| --- | --- |
| PUBMED | (((((((((((((((((((((((((((((((((((((((((((((((cells[MeSH Terms]) OR periodontal ligament[MeSH Terms]) OR tooth[MeSH Terms]) OR tooth injuries[MeSH Terms]) OR tooth avulsion[MeSH Terms]) OR ligament, periodontal[Title/Abstract]) OR ligaments, periodontal[Title/Abstract]) OR periodontal ligaments[Title/Abstract]) OR alveolodental membrane[Title/Abstract]) OR alveolodental membranes[Title/Abstract]) OR membrane, alveolodental[Title/Abstract]) OR membranes, alveolodental[Title/Abstract]) OR alveolodental ligament[Title/Abstract]) OR alveolodental ligaments[Title/Abstract]) OR ligament, alveolodental[Title/Abstract]) OR ligaments, alveolodental[Title/Abstract]) OR teeth[Title/Abstract]) OR injuries, teeth[Title/Abstract]) OR injury, teeth[Title/Abstract]) OR injury, teeth[Title/Abstract]) OR injuries, tooth[Title/Abstract]) OR injury, tooth[Title/Abstract]) OR tooth injury[Title/Abstract]) OR teeth injuries[Title/Abstract]) OR avulsion, tooth[Title/Abstract]) OR avulsions, tooth[Title/Abstract]) OR tooth avulsions[Title/Abstract]) OR avulsed tooth[Title/Abstract]) OR tooth, avulsed[Title/Abstract]) OR dislocation, tooth[Title/Abstract]) OR dislocations, tooth[Title/Abstract]) OR tooth dislocation[Title/Abstract]) OR tooth dislocations[Title/Abstract]) OR tooth luxation[Title/Abstract]) OR luxation, tooth[Title/Abstract]) OR luxations, tooth[Title/Abstract]) OR tooth luxations[Title/Abstract]) OR avulsion[Title/Abstract]) OR dental trauma[Title/Abstract]) OR avulsed teeth[Title/Abstract]) OR periodontal ligament cells[Title/Abstract]) OR trauma[Title/Abstract]) AND storage media[Title/Abstract]) OR transport media[Title/Abstract]) OR storage medium[Title/Abstract]) AND hanks balanced salt solution[MeSH Terms]) OR hanks' balanced salt solution[Title/Abstract]) OR save-a-tooth (salt) solution[Title/Abstract] |
| SCOPUS | TITLE-ABS-KEY(cells) OR TITLE-ABS-KEY("periodontal ligament") OR INDEXTERMS(tooth) OR TITLE-ABS-KEY("tooth injuries") OR INDEXTERMS("tooth avulsion") OR TITLE-ABS-KEY("ligament, periodontal") OR TITLE-ABS-KEY("ligaments, periodontal") OR TITLE-ABS-KEY("periodontal ligaments") OR TITLE-ABS-KEY("alveolodental membrane") OR TITLE-ABS-KEY("alveolodental membranes") OR TITLE-ABS-KEY("membrane, alveolodental") OR TITLE-ABS-KEY("membranes, alveolodental") OR TITLE-ABS-KEY("alveolodental ligament") OR TITLE-ABS-KEY("alveolodental ligaments") OR TITLE-ABS-KEY("ligament, alveolodental") OR TITLE-ABS-KEY("ligaments, alveolodental") OR TITLE-ABS-KEY(teeth) OR TITLE-ABS-KEY("injuries, teeth") OR TITLE-ABS-KEY("injury, teeth") OR TITLE-ABS-KEY("injury, teeth") OR TITLE-ABS-KEY("injuries, tooth") OR TITLE-ABS-KEY("injury, tooth") OR TITLE-ABS-KEY("tooth injury") OR TITLE-ABS-KEY("teeth injuries") OR TITLE-ABS-KEY("avulsion, tooth") OR TITLE-ABS-KEY("avulsions, tooth") OR TITLE-ABS-KEY("tooth avulsions") OR TITLE-ABS-KEY("avulsed tooth") OR TITLE-ABS-KEY("tooth, avulsed") OR TITLE-ABS-KEY("dislocation, tooth") OR TITLE-ABS-KEY("dislocations, tooth") OR TITLE-ABS-KEY("tooth dislocation") OR TITLE-ABS-KEY("tooth dislocations") OR TITLE-ABS-KEY("tooth luxation") OR TITLE-ABS-KEY("luxation, tooth") OR TITLE-ABS-KEY("luxations, tooth") OR TITLE-ABS-KEY("tooth luxations") OR TITLE-ABS-KEY("avulsion") OR TITLE-ABS-KEY("dental trauma") OR TITLE-ABS-KEY("avulsed teeth") OR TITLE-ABS-KEY("periodontal ligament cells") OR TITLE-ABS-KEY(trauma) AND TITLE-ABS-KEY("storage media") OR TITLE-ABS-KEY("transport media") OR TITLE-ABS-KEY("storage medium") AND TITLE-ABS-KEY(“hanks balanced salt solution") OR TITLE-ABS-KEY("hanks' balanced salt solution") OR TITLE-ABS-KEY("save-a-tooth (salt) solution") |
| WEB OF SCIENCE | TS= (cells OR "periodontal ligament" OR tooth OR "tooth injuries" OR "tooth avulsion" OR "ligament, periodontal" OR "ligaments, periodontal" OR "periodontal ligaments" OR "alveolodental membrane" OR "alveolodental membranes" OR "membrane, alveolodental" OR "membranes, alveolodental" OR "alveolodental ligament" OR "alveolodental ligaments" OR "ligament, alveolodental" OR "ligaments, alveolodental" OR teeth OR "injuries, teeth" OR "injury, teeth" OR "injury, teeth" OR "injuries, tooth" OR "injury, tooth" OR "tooth injury" OR "teeth injuries" OR "avulsion, tooth" OR "avulsions, tooth" OR "tooth avulsions" OR "avulsed tooth" OR "tooth, avulsed" OR "dislocation, tooth" OR "dislocations, tooth" OR "tooth dislocation" OR "tooth dislocations" OR "tooth luxation" OR "luxation, tooth" OR "luxations, tooth" OR "tooth luxations" OR "avulsion" OR "dental trauma" OR "avulsed teeth" OR "periodontal ligament cells" OR trauma) AND TS= ("storage media" OR "transport media" OR "storage medium") AND TS= (“hanks balanced salt solution" OR "hanks' balanced salt solution" OR "save-a-tooth (salt) solution")) |
| THE COCHRANE LIBRARY | cells OR "periodontal ligament" OR "tooth" OR "tooth injuries" OR "tooth avulsion" OR "periodontal ligaments" OR "alveolodental membrane" OR "alveolodental ligament" OR teeth OR "tooth injury" OR "teeth injuries" OR "tooth avulsions" OR "avulsed tooth" OR "tooth dislocation" OR "tooth dislocations" OR "tooth luxation" OR "avulsion" OR "dental trauma" OR "avulsed teeth" OR "trauma" in Title, Abstract, Keywords and "storage media" OR "transport media" OR "storage medium" in Title, Abstract, Keywords and "hanks balanced salt solution" OR "hanks' balanced salt solution" OR "save-a-tooth (salt) solution" in Title, Abstract, Keywords |
| OPENGREY | tooth avulsion AND storage media |
| LILACS | (tw:((tw:(cells)) OR (tw:(periodontal ligament)) OR (tw:(tooth)) OR (tw:(tooth injuries)) OR (tw:(tooth avulsion)) OR (tw:(periodontal ligament$)) OR (tw:(alveolodental membrane$)) OR (tw:(alveolodental ligament)) OR (tw:(alveolodental ligament$)) OR (tw:(teeth)) OR (tw:(tooth injury)) OR (tw:(teeth injuries)) OR (tw:(tooth avulsions)) OR (tw:(avulsed tooth)) OR (tw:(tooth dislocation)) OR (tw:(tooth dislocations)) OR (tw:(tooth luxation)) OR (tw:(tooth luxations)) OR (tw:(avulsion)) OR (tw:(dental trauma)) OR (tw:(avulsed teeth)) OR (tw:(periodontal ligament cells)) OR (tw:(trauma)))) AND (tw:((tw:(storage media)) OR (tw:(transport media)) OR (tw:(storage medium)))) AND (tw:((tw:(hanks balanced salt solution)) OR (tw:(hanks' balanced salt solution)) OR (tw:(save-a-tooth (salt) solution)))) |
| GOOGLE SCHOLAR | "tooth avulsion"+"storage media"+"hanks balanced salt solution" |
