# Supplementary material for: Efficacy of Hank's balanced salt solution compared to other solutions in the preservation of the periodontal ligament. A systematic review and meta-analysis
Source: PLoS One. 2018 Jul 13;13(7):e0200467. doi: 10.1371/journal.pone.0200467 (PMC6044542; doi:10.1371/journal.pone.0200467)
Supplement: S2 Appendix — (DOCX) [file pone.0200467.s003.docx]

S2 Appendix: Criteria considered in Risk of Bias evaluation according to ToxRTool.

| Criteria | Description |  |
| --- | --- | --- |
| **Criteria Group I: Test substance identification** |  |  |
| Was the test substance identified?* | The identification of substance was considered adequate (criteria met) when the chemical names, chemical structure, common names ou CAS or EU names were provided. | |
| Is the purity of the substance given? | The criteria met when purity, orign or descripption of preparation of evaluated storage medium | |
| Is information on the source/origin of the substance given? | The criteria met when the source or suplier of the evaluated storage(s) medium(s) were presented. | |
| Is all information on the nature and/or physico-chemical properties of the test item given, which you deem indispensable for judging the data (see explanation for examples)? | The criteria met when additional information of concentration or origin (plant extracts) of the storage medium were provided | |
| **Criteria Group II: Test system characterisation** |  |  |
| Is the test system described? | The criteria met when the protocol of periodontal ligament's cells obterntion were described | |
| Is information given on the source/origin of the test system? | The criteria met when the origin of teeth were described, including type of teeth and cause of extraction/avulsion | |
| Are necessary information on test system properties, and on conditions of cultivation and maintenance given? | The criteria met when the control of contamination of cells against the different mediums were provided. | |
| **Criteria Group III: Study design description** |  |  |
| Is the method of administration given (see explanations for details)? | The criteria met when the total volume of subtance used, number of included cells and description of full method were provided. | |
| Are doses administered or concentrations in application media given?* | The criteria met when the concentration of the used storage mediums were described. | |
| Are frequency and duration of exposure as well as time-points of observations explained?* | The criteria met when the period of storage of the used mediums were described. | |
| Were negative controls included (give also point, if not necessary, see explanations)?* | The criteria met when the negative controls were used and described. | |
| Were positive controls included (give also point, if not necessary, see explanations)?* | The criteria met when the positive controls were used and described. | |
| Is the number of replicates (or complete repetitions of experiment) given? | The criteria met when the number of replicates were fully described. | |
| **Criteria Group IV: Study results documentation** |  |  |
| Are the study endpoint(s) and their method(s) of determination clearly described? | The criteria met when the method of viability tests were described, including methods of evaluation and substances used. | |
| Is the description of the study results for all endpoints investigated transparent and complete? | The criteria met when the results of all endpoints presented on methods of each study were fully presented. | |
| Are the statistical methods for data analysis given and applied in a transparent manner (give also point, if not necessary/applicable, see explanations)? | The criteria met when a fully description of statistical method were provided and was adequate to the objetive of the evaluated study. | |
| **Criteria Group V: Plausibility of study design and data** |  |  |
| Is the study design chosen appropriate for obtaining the substance-specific data aimed at (see explanations for details)?* | The criteria met when the design of study was adequate. | |
| Are the quantitative study results reliable? | The criteria met when the study present na adequate matching betwenen groups, the variability between negative and positive controls were acceptable or no reason were aplicable to mistrust the numerical data | |
| Final Category | According to sum os points of all qiestions, the final category must be classificated as: | |
|  | 1 (reliable without restrictions): 15-18 final points; | |
|  | 2 (reliable with restrictions): 11-14 points; | |
|  | 3 (not reliable): < 11 or with not all "*" criteria met; | |
|  | 4 (not assignable): Insufficient documentation for analysis | |
